# *A Fatal Instance of the Poisonous Effects of the Oenanthe Crocata* Linn. *or Hemlock Dropwort*

**Published:** 1797

**Authors:** Robert Graves

**Affiliations:** Physician at Dorchester, and Extra Licentiate of the College of Physicians, London.


XXVII. *A fatal Inſtance of the poiſonous Effects of the Oenanthe Crocata* Linn. *or Hemlock Dropwort. Communicated in a Letter to Dr.* Simmons, *by* Robert Graves, M. D. *Phyſician at Dorcheſter, and Extra Licentiate of the College of Phyſicians, London.*

AMONG the numerous poiſonous plants with which the vegetable kingdom abounds, abounds, there are few, perhaps, natives of this country, more virulent and ſpeedy in their effects, than that which gave birth to the following melancholy incident; in confirmation of which, indeed, too many fatal examples are to be found, ſcattered in the writings of medical men. Againſt ſo deſtructive an enemy to mankind, therefore, it behoves us to be particularly on our guard; and every unhappy inſtance wherein the contrary takes place ought not, in my opinion, to be ſuffered to paſs wholly unrecorded. It is only from being made acquainted with the errors or defective caution of others, that we, in many caſes, become mindful to keep clear of ſuch ourſelves.

A young woman, about twenty-four years of age, ſervant to a lady in this neighbourhood, having been recommended by a medical practitioner of eminence, to take the juice of the water-parſnep for an eruptive complaint in her face, became exceedingly deſirous of trying it in the month of May laſt. The gardener of the family was applied to, and earneſtly entreated by her to collect the plant; but from its not being ſufficiently known to him, he at firſt heſitated, very properly, to comply with her requeſt. It happened, that in a moiſt watery ditch or brook, within a ſhort diſtance of the houſe where ſhe lived, a large quantity of hemlock dropwort was growing. This unfortunately ſhe diſcovered, and conceiving, both from its ſmell and appearance, to be the herb which ſhe was in queſt of, ſhe communicated the ſame to the gardener, with an expreſs declaration, that if he would not gather ſome for her, ſhe would undertake to do it herſelf. The gardener being thus forcibly ſolicited, and not aware of the virulent and deadly nature of the plant which he was about to furniſh her with, was at length brought to compliance.

On the morning of the 19th of the ſame month, being plentifully ſupplied with the whole plant, ſhe took the roots of it, bruiſed them herſelf, and expreſſed from them, as it was ſuppoſed, juice to the amount of two table ſpoonfuls at leaſt, which ſhe ſwallowed. Scarcely had more than ten or fifteen minutes elapſed afterwards, before ſhe complained of great giddineſs, accompanied with vaſt uneaſineſs and ſickneſs at ſtomach, though not ſuch as to be ſucceeded by vomiting; convulſions likewiſe ſpeedily ſupervened, and ſhe became entirely deprived of her ſenſes.

It was about half an hour after this, when I

was was called to her affiftance. At that period the convulfive paroxyfms were become not only ftronger, but recurred in quicker fucceffion; all fenfibility was deftroyed; the mouth firmly open, with fcarce any pulfe to be felt at the wrift, and a coldnefs had feized the extremities. Prior to my feeing her, repeated draughts of milk and oil had been got down, agreeably to the advice of Mr. Arden, a judicious and experienced furgeon of this place, but without the leaft apparent effect, or ficknefs, fucceeding. Wifhing, therefore, if poffible, notwithftanding the truly hopelefs ftate of the cafe, to folicit a difcharge of the poifon as the moft effectual ftep towards affording relief, an emetic was immediately fent for; but though it arrived in the fhort fpace of ten or twelve minutes, fhe did not furvive even to take it. Befides the repeated draughts of milk and oil above mentioned, between two and three ounces of vinegar alfo were forced down, at my direction, from an idea that this acid might act fomewhat as a corrective.

I fhall clofe this cafe with a very neceffary caution, and which I think cannot be too ftrongly impreffed on the minds of medical men. Though that fpecies of the water-parfnep,

(*Sium nodiflorum* Linn.) directed by the London college, bears but little resemblance indeed to the hemlock dropwort; yet from the fatal error here related, joined to a similar one, which happened also in this county but a few years since*, practitioners, I conceive, ought on no account, whenever they should deem it proper to recommend the former plant, to trust to unskilful people to gather it. Both plants are to be found frequently inhabiting the very same moist, watery situations; and the ignorant and unwary are as likely to be imposed upon by the latter, as to have their choice determined, or attention drawn by the former.

*Dorchester,*

October 21, 1796.

* London Medical Journal, Vol. 5, p. 192.

CATALOGUE

